# A Novel Nano-Scale Biosensor for Measuring Hemoglobin Oxygen Saturation Using Carbon Quantum Dots

**DOI:** 10.3390/mi16111261

**Published:** 2025-11-06

**Authors:** Jeehyun Lee, Xuan Ru Liew, Justin Kok Soon Tan, Sangho Kim

**Affiliations:** Department of Biomedical Engineering, National University of Singapore, Singapore 117583, Singapore; e1091881@u.nus.edu (J.L.); xuanru@u.nus.edu (X.R.L.); j.tan@nus.edu.sg (J.K.S.T.)

**Keywords:** hemoglobin oxygen saturation, carbon quantum dots, fluorescence, photoluminescence quenching

## Abstract

Hemoglobin oxygen (HbO_2_) saturation is a critical biomarker in patient care, yet conventional measurement approaches are often costly and require extensive calibration. To address these limitations, the present study proposes a novel biosensor derived from paper-based carbon quantum dots (CQDs) fabricated through a one-step thermal treatment. CQDs are carbon-based nanoparticles renowned for their excellent biocompatibility, low toxicity, thermal stability, and remarkable optical properties. To quantify HbO_2_ saturation, we exploit their photoluminescence, which enables photoinduced electron transfer and fluorescence quenching with hemoglobin. Our results demonstrated that the peak fluorescence intensity of CQDs shows a strong linear correlation with HbO_2_ saturation. Variations in HbO_2_ saturation levels were achieved with sodium dithionite and determined using Winterbourn’s equations. Our CQD-based HbO_2_ saturation measurements closely agreed with those obtained from conventional spectrophotometric analysis. Thus, this investigation highlights the potential of CQDs as a biosensor for effective HbO_2_ saturation measuring without extensive calibration.

## 1. Introduction

Hemoglobin oxygen (HbO_2_) saturation is an essential element in patient care, as red blood cells tightly regulate and determine blood flow in the circulatory system. The lack of measurement of HbO_2_ saturation levels may result in undetected hypoxia, potentially leading to acute adverse events [1]. The normal HbO_2_ saturation level ranges from 95% to 100% and values under 90% are considered a serious deterioration in status with a high risk of hypoxia. Those under 70% are in a life-threatening state [2]. Hypoxia occurs when oxygen is insufficient at the tissue level and hypoxemia occurs when arterial oxygen tension is below normal [3]. It is generally known that hypoxemia can lead to hypoxia. Clinically, there is no set standard HbO_2_ saturation level where hypoxemia occurs [4]. Thus, it is significant to be able to measure HbO_2_ saturation over a wider range. Current methods for HbO_2_ saturation detection include pulse oximetry, near-infrared spectroscopy, hyperspectral imaging, photoacoustic imaging, and arterial blood gas analysis. However, these existing techniques exhibit a trade-off between cost and accuracy, achieving one at the expense of the other. Moreover, it has been recognized that the dynamic measurement of HbO_2_ saturation changes is challenging with current state-of-the-art technology due to the complexity of calibration and detection protocols. In this study, we designed an innovative nano-sensor using carbon quantum dots (CQDs) to detect the temporal variations of HbO_2_ saturation level in a blood sample with the goal of achieving greater reliability and simplicity.

There have been some nanostructures used to indirectly measure HbO_2_ saturation state or levels. According to a previous study [5], gold nanoparticle-covered SiO_2_ substrates can detect hemoglobin’s oxygenation-dependent vibrational markers, such as Fe-O_2_ stretching and heme modes, to determine oxygenated and de-oxygenated states of hemoglobin using Raman spectroscopy. Furthermore, there are nanostructures using metal-ligands such as Ruthenium or Porphyrin platinum complexes to measure the partial pressure of oxygen in blood. Then, these partial pressure values could be correlated to HbO_2_ saturation levels following the oxygen-hemoglobin dissociation curve [6]. An electrical sensor using TiS_2_ nanosheets and graphene-based optical sensors was also reported to measure partial pressure of oxygen to determine HbO_2_ saturation [7]. Such indirect methods of measuring HbO_2_ saturation using nanostructures highlight the necessity for a direct measurement of HbO_2_ saturation. Carbon is universally present in all organic matter, and carbon nanoparticles are a nanomaterial formed from pure carbon. In the class of carbon nanoparticles, CQDs are those with a size of less than 10 nm. The properties of CQDs include low toxicity, good biocompatibility, biodegradability, and tunability, attracting attention in the biomedical application field. In addition, CQDs demonstrate intrinsic state luminescence and the quantum confinement effect, a phenomenon where nanoscale energy carriers follow scaling laws based on their size variation [8,9]. Recently, we successfully demonstrated a fabrication method for CQDs from cellulosic paper for biosensor applications [10]. The interwoven fibers of paper provide a large surface area for the efficient fabrication of CQDs. With such a synthetic advantage, we found that the fluorescence intensity (FI) of CQDs quenches in the presence of Fe^3+^ ions and cell-free hemoglobin, consistent with photo-induced electron transfer from oxygen-rich surface groups on CQDs and metal ions. Thus, fluorescence quenching is selective depending on the strength of binding affinities between metal ions and the surface groups on the fluorescent particles.

Building on our earlier findings, we sought to extend their application to the detection of HbO_2_ saturation. Our previous results demonstrated that CQDs can be used to detect cell-free hemoglobin in blood bags and monitor blood storage conditions, thereby improving blood bank inventory management [10]. We also observed a degree of variability in the level of fluorescence quenching of CQDs across different blood samples, which we attributed to differences in HbO_2_ saturation levels. Accordingly, we hypothesized that CQDs are capable of detecting dynamic changes in HbO_2_ saturation levels. To test this, HbO_2_ saturation levels were controlled using sodium dithionite as an oxygen scavenger, with reoxygenation occurring over time as oxygen re-entered the solution. Such varying HbO_2_ saturation values were quantified by optical spectroscopy at selected wavelengths, which were then used to evaluate the optical responses of our CQDs. Finally, the performance of the CQD-based sensor in sensing HbO_2_ saturation levels was validated by comparison with an existing method.

## 2. Materials and Methods

### 2.1. Carbon Quantum Dot (CQD)

**Figure 1:**
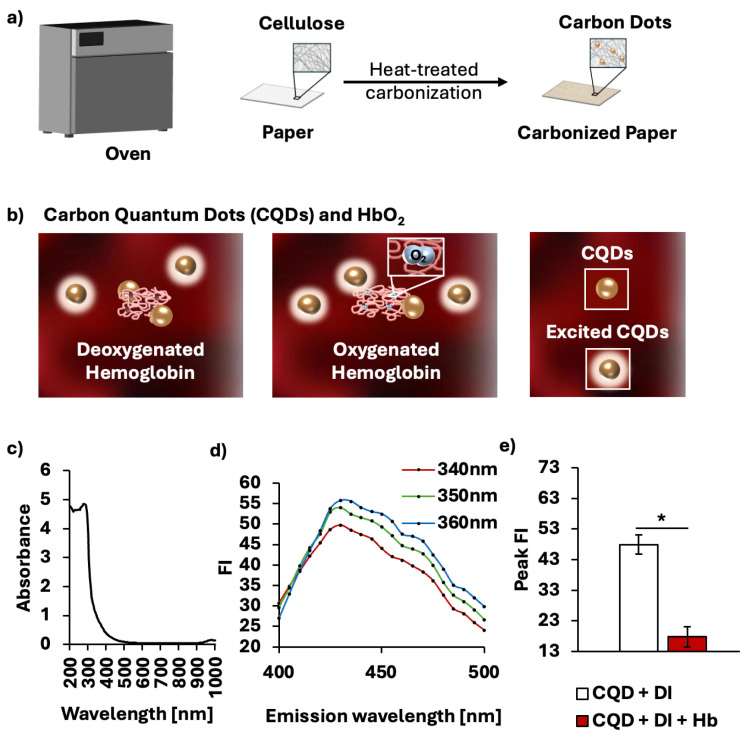
Synthesis of CQDs and characterization of CQDs in interaction with hemoglobin. (**a**) Schematic illustration showing one-step thermal fabrication to produce in situ CQDs on paper. (**b**) Interactions of CQDs and hemoglobin with different oxygenated states. (**c**) Absorbance spectrum of CQDs from 200 to 1000 nm. (**d**) Emission spectrum of CQDs with excitation wavelength at 340, 350, 360 nm. (**e**) Average peak FI with and without cell-free hemoglobin, showing that the fluorescence quenching of CQDs due to hemoglobin is statistically significant. * *p* < 0.001.

We fabricated CQDs from paper through a one-step thermal treatment using a laboratory-grade drying oven (SLN 15, Pol-Eko, Wodzisław Śląski, Poland) as reported recently [10]. Cellulosic paper with a thickness of 180 μm and a pore size of 11 μm (Whatman^®^ Grade 1, GE Healthcare, Buckinghamshire, UK) was laid flat on a stainless-steel tray and baked at 260 °C for 10 min. The temperature of 260 °C was chosen due to its low fluorescence emission variability. To isolate CQDs from the baked cellulose paper, the paper was cut into strips of 800 mg and resuspended in 4 mL of deionized (DI) water. The mixture was then agitated using a vortex mixer (Orbital Vortex Mixer, AIT biotech, Singapore) for 2 min. The excess supernatant containing CQDs was extracted, and the vortex-extraction step was repeated three more times. To remove large cellulose fibers, the supernatant was centrifuged at 15,000× *g* for 10 min (Sartorius 1-14, Göttingen, Germany), after which the supernatant was filtered through a 0.22-μm microporous membrane (Minisart^®^ High Flow, 6 Sartorius, Goettingen, Germany). Figure 1a provides a brief description of the fabrication procedure. The varying concentrations of CQDs were prepared by oven-drying at 70 °C to accelerate natural evaporation. The quenching property of CQDs to detect the HbO_2_ saturation levels is described in Figure 1b. The oxygenation level of hemoglobin appears to influence the extent of fluorescence quenching of CQDs in presence of hemoglobin, leading to variability in FI of CQDs. Lower oxygen levels correspond to greater quenching and thus lower absolute FI of CQDs. Yet, the exact mechanism by which oxygen modulates CQD-hemoglobin remains unclear. The absorbance spectrum of CQDs is shown with a peak absorbance at 275 nm (Figure 1c), supporting π– π* transitions of aromatic sp^2^ domains in the C=C bonds in CQDs [10]. After the peak absorbance, there is a sharp decrease up to 315 nm, followed by a plateau to zero. These near-zero absorbance values from 550 to 650 nm ensure that CQDs do not interfere with the quantification of HbO_2_ saturation. CQDs fabricated from cellulosic paper exhibit a peak FI level at 360 nm (Figure 1d). Figure 1e verifies the ability of CQDs to quench in the presence of hemoglobin (*p* < 0.001). The solution containing CQDs in DI water exhibited a peak FI of 47.88 ± 3.13, whereas the addition of hemoglobin reduced it to 17.76 ± 3.33.

### 2.2. Characterization of CQDs

Detailed information on the characterization of paper-derived CQDs can be found in our recent study [10]. Here, we summarize the materials science data for our CQDs. The morphological characterization of paper-derived CQDs through dynamic light scattering (DLS) confirmed a size distribution within the range of 2.3 to 3.6 nm, with an average diameter of 2.9 ± 0.5 nm, consistent with typical CQD dimensions. Based on FTIR analysis, broad O-H stretching at 3315 cm^−1^ demonstrates the existence of hydroxyl (-OH) and carboxyl (-COOH) groups. The peak at 2889 cm^−1^ shows a C-H stretching vibration. The peak at 1631 cm^−1^ indicates C=C bonds, while the peak at 1024 cm^−1^ indicates C-O stretching. Additionally, cellulose-related bands are observed at 892, 1109, and 1155 cm^−1^. Moreover, XPS analysis supported the FTIR findings through C1s peaks at 283.0 eV (C=C), 284.7 eV (C-C), and 286.1 eV (C-O), as well as the O1s peak at 532.5 eV (C-O). Raman spectroscopy showed peaks between 1095 and 1122 cm^−1^, the axial deformation of C-O, matching cellulose bands. Our CQDs showed significant fluorescence quenching by Fe^3+^ and Cu^2+^, which can be attributed to strong binding between ions and oxygenated groups (-OH, -COOH). In addition, the strongest quenching was observed by hemoglobin, as also confirmed in this study.

### 2.3. Blood Sample Preparation

Fresh porcine whole blood was collected from the abattoir (Primary Industries, Singapore) in 3.2% sodium citrate. Porcine blood was centrifuged at 5000× *g* for 10 min (Megafuge 8, Thermo Scientific, Waltham, MA, USA). The plasma and buffy coat were removed from the sample. Packed red blood cells (PRBCs) were prepared by washing three times with 1X phosphate-buffered saline solution. Then, the PRBC was suspended in DI water at 40% hematocrit to induce lysis. To confirm that the RBCs are fully lysed, the RBC solution was ultrasonicated. The total hemoglobin concentration was then measured using a hemoglobin analyzer (Hb 301, Hemocue, Ängelholm, Sweden).

### 2.4. HbO_2_ Saturation Level Control

To achieve different HbO_2_ saturation levels, we used sodium dithionite (Na_2_S_2_O_4_) (157953, Sigma Aldrich, Darmstadt, Germany) to remove oxygen from the sample. To ensure the stability of the sodium dithionite solution, a high volume of solution was freshly prepared daily: a varying amount of solid sodium dithionite was added to a 15-mL centrifuge tube containing 10 mL of DI water. Then, all samples were sealed properly to avoid the uncontrolled introduction of oxygen from the air. Dithionite induces hemoglobin to release oxygen by lowering the oxygen concentration in the solution. When used at a proper concentration, sodium dithionite can reoxygenate the blood sample over time and reverse its effects [11]. For validation of its effects, an electrochemical oxygen gas sensor probe (OX-10-17161, Unisense, Aarhus, Denmark) was utilized.

### 2.5. HbO_2_ Saturation Level Measurement

In all experiments, a hemoglobin concentration of 0.25 g dL^−1^ was chosen to ensure a good balance between minimizing noise and reliably detecting the oxygenation absorbance peaks. To quantify HbO_2_ saturation, the concentration of oxyhemoglobin (OxyHb) and deoxyhemoglobin (DeoxyHb) was determined using equations derived from Winterbourn extinction coefficients as follows [12,13,14]:

$$\left[Oxy\;Hb\right]=-75.78\;{OD}_{560}+103.16\;{OD}_{576}-38.39\;{OD}_{630}$$

$$\left[Deoxy\;Hb\right]=132.6{OD}_{560}+74.10{OD}_{576}-68.33\;{OD}_{630}$$

Then, HbO_2_ is determined by the following equations:

$${HbO}_{2}=\;\frac{\left[OxyHb\right]}{\left[OxyHb\right]+[DeoxyHb]}$$

Oxyhemoglobin is the form of hemoglobin with bound oxygen molecules, while deoxyhemoglobin is the form of hemoglobin with no oxygen molecules. Each form of hemoglobin was calculated using the Winterbourn equation, based on absorbance values at reported wavelengths (560, 576, and 630 nm). Absorbance values of samples were obtained through a multimode microplate reader (Varioskan LUX, Thermo Fisher Scientific, Waltham, MA, USA). At these wavelengths, one component is at maximal absorbance with minimal interference from others, ensuring accuracy of measurements [13,14]. Then, the HbO_2_ saturation level was subsequently determined by the ratio of oxyhemoglobin to the sum of oxyhemoglobin and deoxyhemoglobin, yielding a value ranging from 0 to 1.

### 2.6. Validation of CQDs for Oxygen Sensing

We have demonstrated that the peak FI of CQDs changes with the saturation levels of HbO_2_. As the saturation of HbO_2_ increased, there was a corresponding increase in the peak FI observed in a solution containing 37.5 μL of 0.25 g dL^−1^ hemoglobin, 75.0 μL of CQDs and 37.5 μL of sodium dithionite solution. To measure the peak FI of hemoglobin solutions with CQDs added at varying HbO_2_ saturation levels, individual wells of a transparent 96-well plate (650901, Greiner Bio-One, Kremsmünster, Austria) were sequentially analyzed. The microplate reader (Varioskan LUX, Thermo Fisher Scientific, Waltham, MA, USA) measures the absorbance for HbO_2_ saturation and fluorescence at an excitation wavelength of 360 nm to find the peak FI. To validate our measurements of HbO_2_ saturation, we employed a spectrophotometric method adapted from a pulse oximetry-based method, incorporating the Beer-Lambert Law and the molar extinction coefficient [15,16].

### 2.7. Statistical Analysis

Statistical analyses were performed in RStudio (version 2024.12.1+563; Posit, Boston, MA, USA). All measurements were performed in triplicate, and results are presented as mean ± standard deviation (SD). Sensitivity testing was performed through analysis of covariance (ANCOVA), and other parametric samples were assessed through a two-tailed *t*-test with unequal variance. Statistical significance was indicated by a *p*-value less than 0.05.

## 3. Results and Discussion

**Figure 2:**
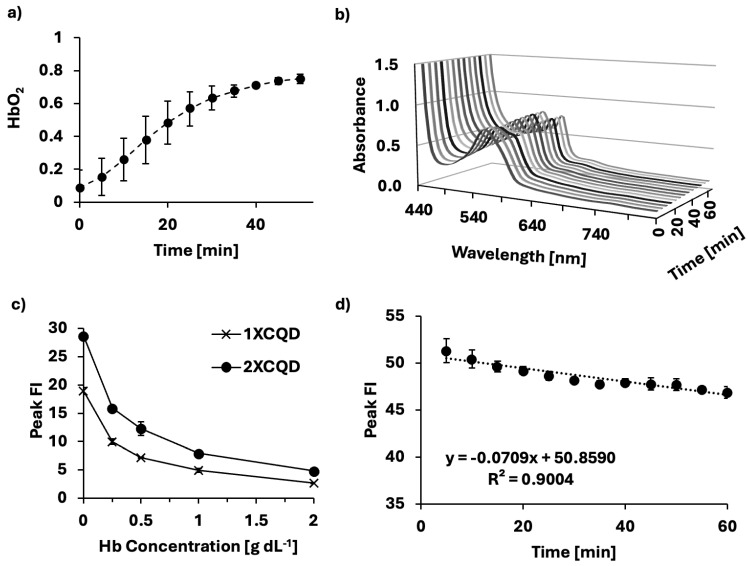
Methodology for producing a biosensor of HbO_2_ saturation levels. (**a**) Sodium dithionite was used to control different hemoglobin oxygen saturation levels over 1 h at 37 °C. (**b**) Absorbance spectrum of porcine blood samples during the process of oxygenating, measured at 5-min intervals for 1 h from deoxidized states to oxidized states (deoxidized states achieved using sodium dithionite). (**c**) Relationship between different concentrations of CQDs and different hemoglobin concentrations demonstrating dose-dependent quenching of CQDs due to hemoglobin. (**d**) Photobleaching effect throughout the experiment (Slope = −0.0709, SD = 0.0075, *p* < 0.005).

The oxygen scavenging ability of sodium dithionite was validated using an electrochemical oxygen gas sensor probe that measures voltage running through the solution in real-time after calibration. At the initial state (t = 0), after the addition of the sodium dithionite solution to the hemoglobin solution, hemoglobin was fully deoxygenated and reoxygenated over time (Figure 2a). HbO_2_ saturation can be spectroscopically distinguished in the Q-band region. At t = 0, the absorbance spectrum in Figure 2b exhibited a single Q-band peak at 555 nm, characteristic of deoxygenated hemoglobin [12,17], correlating to a low HbO_2_ saturation. During the measurement, the absorbance spectrum changed, showing two peaks at 540 and 575 nm, indicating that the hemoglobin solution had been successfully reoxygenated, characteristic of oxygenated hemoglobin. The effect of hemoglobin concentration on the peak FI emitted by two different concentrations (1X and 2X) of CQDs was investigated. The peak FI emission shows a similar increase with decreasing hemoglobin concentration (Figure 2c) at both CQD concentrations. While 2X concentration began with a higher peak FI compared to 1X concentration, both progressively quenched with increasing hemoglobin concentrations. As CQDs are constantly excited whilst measuring different HbO_2_ saturation levels, the photostability of CQDs under prolonged exposure to UV light was investigated (Figure 2d). When CQDs are excited, the fluorophore is structurally unstable and is susceptible to slight degradation. We found the photobleaching effect on CQDs to be significant (*p* < 0.005) with a negative linear relationship (R^2^ = 0.9004). In this study, HbO_2_ saturation levels were controlled in a time-dependent manner using sodium dithionite, necessitating the excitation of CQDs multiple times within a short timeframe. Thus, to minimize the influence of photobleaching on the interpretation of CQDs as a biosensor, the loss of FI due to photobleaching was compensated during the data analysis.

**Figure 3:**
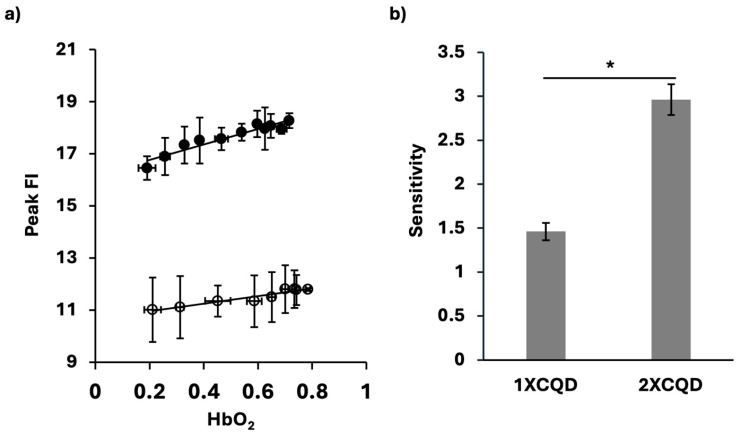
A biosensor of HbO_2_ using CQDs for peak FI. (**a**) Peak FI of CQDs at different HbO_2_ saturation levels using 1XCQD concentration (○) and 2XCQD concentration (●). For 1XCQD, *y* = 1.4573*x* + 10.663, R^2^_1XCQD_ = 0.9149, *p* < 0.0001. For 2XCQD, *y* = 2.9587*x* + 16.174, R^2^_2XCQD_ = 0.9125, *p* < 0.0001. Each data point reflects measurements from samples (n = 40 for 1XCQD and n = 44 for 2XCQD). (**b**) HbO_2_ saturation level sensitivity based on the slope. Using 2XCQD concentration showed a higher sensitivity of 2.96 compared to using 1XCQD of 1.46. * *p* < 0.001. All the tests were conducted at 37 °C.

As shown in Figure 3a, there was a strong linear relation between the HbO_2_ saturation levels and the peak FI of CQDs in the presence of hemoglobin. This linear increase in peak FI corresponded to increasing HbO_2_ saturation, highlighting the potential of peak FI as a biosensor for oxygenation levels of hemoglobin in the samples. To further test the influence of the concentration of CQDs in this measurement, 1X and 2X CQD concentrations were examined. Both concentrations resulted in a high correlation (R^2^_1XCQD_ = 0.9149, R^2^_2XCQD_ = 0.9125). Compared to the case of 2XCQD, the 1XCQD results showed higher peak FI variability (Figure 3a). Furthermore, there was a significant increase in the sensitivity of HbO_2_ saturation detection with an increase in the concentration of CQDs (Figure 3b). Using 2XCQD for the HbO_2_ sensor, we found the higher sensitivity of 2.96 ± 0.18, whereas with 1XCQD, 1.49 ± 0.097. The linear detection range of HbO_2_ saturation was from 0.2110 to 0.7851. The limits of detection and quantification (LOD and LOQ) were determined to be 0.18 and 0.58, respectively (n = 4). The following formulas were used: LOD = (3 × SD_HbO2 = 0.21101_)/slope and LOQ = (10 × SD_HbO2 = 0.21101_)/slope, where SD_HbO2 = 0.21101_ is the standard deviation at HbO_2_ of 0.21101. HbO_2_ of 0.21101 is chosen as it is the lowest hemoglobin oxygen saturation level measured. Lower HbO_2_ levels could not be achieved since oxygen from ambient air was impulsively introduced during spectroscopic measurement for peak FI after deoxygenation with sodium dithionite.

**Figure 4:**
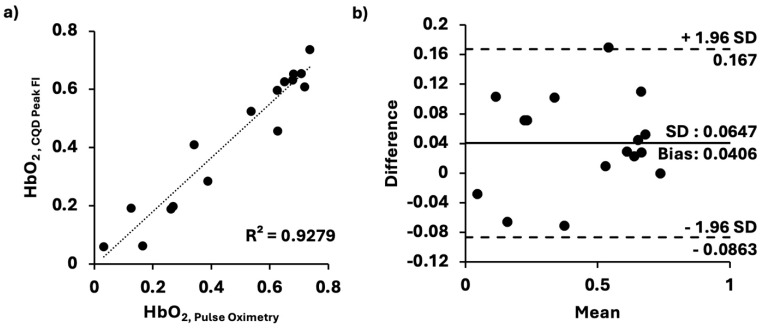
Comparison between our and conventional pulse oximetry methods. (**a**) HbO_2_ saturation levels determined by our method and pulse oximetry; linear relationship (*y* = 0.9255*x* − 0.0054, SD = 0.0658, *p* < 0.0001), closely approximating an identity function. (**b**) Bland–Altman plot comparing the two methods. All the data were evaluated at 37 °C on a clear 96-well plate under the same conditions.

The result confirmed that our CQD method can quantify dynamic changes in hemoglobin saturation. To test the accuracy of our measurements, we compared ours with those obtained using an adapted pulse oximetry method. For this comparison test, the 1XCQD concentration was selected to assess its minimal accuracy in our measurement of HbO_2_ saturation levels. As previously stated, the different levels of HbO_2_ saturation were time-dependent as the blood sample re-oxygenated over time after the addition of sodium dithionite solution. Samples for both our CQDs and the conventional pulse-oximetry methods were exposed to air with identical surface areas, thereby controlling the rate of hemoglobin oxygenation over time. In Figure 4, the HbO_2_ saturation level values (n = 16) obtained from the adapted pulse oximetry method were compared with those from our CQD method (n = 16). Our peak FIs of CQDs in hemoglobin solution results were in good agreement with those obtained from the conventional system, exhibiting a strong linear correlation (R^2^ = 0.9279) (Figure 4a). Bland–Altman analysis (Figure 4b) showed a mean bias of 0.0406 and an SD of 0.0647 relative to the conventional method. The upper and lower limits of agreement were 0.167 and −0.0863, respectively. Hence, it demonstrated that our CQDs for HbO_2_ saturation level sensing would be as accurate as the conventional method.

The capacity of CQDs as a novel biosensor is closely related to the synthesis method used. Broadly speaking, CQD synthesis routes can be classified into two main approaches: “top-down” and “bottom-up.” Our CQDs were obtained via the “top-down” approach, in which exfoliation and cutting of large carbon materials are performed after carbonization. Our CQDs were fabricated from cellulosic paper through thermal treatment at 260 °C for 10 min, a process that is quick, simple, and cost-efficient. Moreover, this process avoids hazardous chemicals and the use of high energy, unlike the traditional method. Thus, such simplicity marks a significant advancement toward the biosensing potential of CQDs to detect HbO_2_ saturation levels. While several existing methods are available to quantify HbO_2_ saturation (Supplementary Table S1), there is a clear need for a rapid yet reliable method to detect the dynamic changes in HbO_2_ saturation. Pulse oximetry is often regarded as the fifth vital sign, making it a widely accepted and valid diagnostic tool [18,19]. However, its accuracy to assess oxygenation drops to 83.2% when peripheral oxygen saturation falls below 90% [20]. Detecting low HbO_2_ saturation levels is crucial for determining whether to initiate or increase oxygen therapy. Hyperspectral imaging provides high spatial resolution of skin and tissue oxygen saturation [21,22]. Yet, it comes at a significantly high cost and requires a substantial amount of time to perform the analysis [21,23,24]. Moreover, all the existing methods require a complex measurement procedure and periodic calibration to ensure accuracy and reliability. On the other hand, our CQDs enable the development of a nanosensor to rapidly detect the HbO_2_ saturation level using a microplate reader. Since sodium dithionite induces time-dependent deoxygenation and reoxygenation, continuous excitation of CQDs over a short period was required in our experimental setup. However, in a clinical setting, such continuous excitation of CQDs over a short period would not be necessary. In addition, our CQDs show no significant change in FI after being stored for 42 days, demonstrating a good photostability [10]. Unlike other methods that rely on multiple absorbance wavelengths, our CQD-based approach benefits from a straightforward linear relationship between peak FI and HbO_2_ saturation levels.

To generate controlled oxygenation states for testing, we used sodium dithionite, which reliably induces deoxygenation in hemoglobin, followed by gradual reoxygenation upon exposure to ambient air [25,26,27]. This reproducible cycle makes dithionite one of the most practical reagents for generating varying HbO_2_ states in vitro. However, it should be noted that sodium dithionite may act as a potent reducing agent, lowering the partial oxygen pressure, when it is added to the hemoglobin solution. This reduction generates hydrogen peroxide and other unstable oxidation products, such as the superoxide anion radical and the reactive sulfur dioxide radical [28]. These byproducts may introduce uncontrolled and unknown reactions that interfere with the heme-binding response [29]. Although such species can, in principle, interfere with hemoglobin and complicate spectral interpretation, their effects are generally short-lived and transient. Notably, a classic study by K. Hamada et al. [30] reported that, under acidic conditions or in the presence of cyanide, transient intermediates with absorbance peaks at 405 and 417 nm were observed. However, our conditions—cell-free porcine hemoglobin in DI water without cyanide—differed from those reports. Moreover, these wavelengths do not overlap with the analytical wavelengths (560, 576, 630 nm) used for HbO_2_ determination in this study. Hence, our measurements of HbO_2_ saturation levels are unlikely to be affected by the byproducts.

CQD-based biosensor, although it remains in vitro, can extend toward clinical use. The normal hemoglobin level for males is 14 to 18 g dL^−1^ and for females is 12 to 16 g dL^−1^ [31]. Each microplate well for measuring HbO_2_ will require a sample amount of 37.5 μL with a concentration of 0.25 g dL^−1^. Owing to the low volume required for measuring HbO_2_ level, a single drop of blood (35 μL) from the patient would allow testing in ~60 wells.

## 4. Conclusions

Our study investigated the potential of a one-step, heat-based synthesis of CQDs in the development of a carbon-based oxygen sensor. There was a strong linear correlation between the HbO_2_ saturation levels and the peak FI of CQDs in the presence of hemoglobin. Compared to conventional methods for sensing HbO_2_ saturation, our CQD sensor provided rapid detection capability through a microplate reader and stable measurements of dynamic changes in HbO_2_ saturation ranging from 0.21 to 0.79. The limits of detection and quantification of our CQD sensor were 0.18 and 0.58, respectively. Demonstrating reliability of CQD measurements, comparison between our CQD method and the conventional pulse oximetry method resulted in a strong linear relationship (n = 16, R^2^ = 0.9279,) and Bland–Altman analysis gave a mean bias of 0.0406 with SD = 0.0647, yielding 95% limits of agreement. With further optimization, this approach could be extended to clinical use under various physiological and pathological conditions.

## Data Availability

The raw data supporting the conclusions of this article will be made available by the authors on request.

## Supplementary Materials

The following supporting information can be downloaded at: https://www.mdpi.com/article/10.3390/mi16111261/s1, Supplementary Table S1: Methods of measuring HbO_2_. References [18,19,20,21,22,23,32,33] are cited in the supplementary materials.

## Abbreviations

CQDs	Carbon Quantum Dots
HbO_2_	Hemoglobin oxygen
FI	Fluorescence Intensity
DI	Deionized (water)
PRBC	Packed Red Blood Cell
OxyHb	Oxyhemoglobin
DeoxyHb	Deoxyhemoglobin
SD	Standard Deviation
ANCOVA	Analysis of Covariance
R^2^	Coefficient of determination
*p*	*p*-value
Na_2_S_2_O_4_	Sodium Dithionite
UV	Ultraviolet
