# Supplementary material for: A Novel Nano-Scale Biosensor for Measuring Hemoglobin Oxygen Saturation Using Carbon Quantum Dots
Source: Micromachines (Basel). 2025 Nov 6;16(11):1261. doi: 10.3390/mi16111261 (PMC12654390; doi:10.3390/mi16111261)
Supplement: Supplementary file 1 [file micromachines-16-01261-s001.zip › micromachines-3953662-supplementary.pdf]

Supplementary File S1: Photobleaching Effect Compensation in Fluorescence Measurements

In Figure 2d, the fluorescence intensity (FI) of the carbon quantum dots (CQDs) decreased linearly over time under continuous excitation, with a slope of -0.0709 min^{-1}. To compensate for photobleaching effect, the percentage decrease in FI was calculated from the fitted linear regression.

<u>Sample Calculation:</u>

According to Figure 2d, the percentage decrease in FI from 0 to 5 minutes is

$$\frac{(50.8590)\ -\ (\,-0.0709 \times 5\ +\ 50.8590)}{50.5890} \times 100 = 0.6970\ \%$$

Then, 0.6970% would be a compensated factor.

Supplementary Table S1: Methods of measuring HbO_2

Method	Description	Pros	Cons
CQD nanoparticles	Detects changes in fluorescence intensity of carbon quantum dots (CQDs) upon interaction with hemoglobin.	• Rapid optical response.<br>• Direct Measurement of HbO_2.<br>• Simple detection set up.<br>• Wider range of detection.	• Requires extracted blood samples (in vitro).<br>• CQDs are not reusable due to irreversible interaction with hemoglobin.<br>• Exact mechanism of oxygen-dependent quenching remains under investigation.
Pulse Oximetry	Uses Beer-Lambert law of light absorption, by emits 2 light pulse of different wavelengths (660, 940 nm) and measures the changes over time [1, 2].	• Non-invasive, inexpensive, and portable.<br>• User-friendly and widely accepted [1].	• Measures only arterial blood SpO_2 [2, 3].<br>• Cannot accurately assess oxygenation when SpO_2 below 90% [4].
Near-infrared spectroscopy (NIRS) [3]	Measure tissue oxygenation in cerebral and muscle tissue— arterial, venous and capillary. Different absorption spectrum of OxyHb and DeoxyHb.	• Non-invasive.<br>• Painless.	• Other biomolecules may be measured simultaneously.<br>• Expensive.<br>• Requires very sensitive detector.
Hyperspectral imaging	Simultaneously provides information about the different tissue constituents and their spatial distribution via spectral absorption [5]. OxyHb peaks at 541 and 576 nm and DeoxyHb peaks at 555 nm.	• Enables in vivo tissue analysis and offers higher spatial resolution [5, 6].<br>• Allows the creation of comprehensive oxygen saturation maps for the entire surgical area.<br>• Non-invasive.	• Tissue heating due to the intense illumination over the same area [5-7].<br>• Spatial scanning faces challenges in co-registration, low acquisition rates, and lack of compactness.<br>• Spectral scanning with tunable filters has limitations in light transmittance and spectral purity.
Photoacoustic imaging [8]	When absorbed photon light is converted into heat, inducing a thermal-elastic expansion in the tissue and emitting acoustic waves. OxyHb and DeoxyHb absorb light differently at different wavelength, leading to different acoustic signal.	• Creates non-contact high spatial-temporal resolution images.<br>• Acoustic waves are less scattered in tissues than photons.	• Cannot measure blood oxygenation in deep tissue beyond ~1 mm.<br>• Signal amplitude is proportional to local optical absorption.<br>• Wavelength-dependent optical attenuation limits the ultimate imaging depth and mixes the measurements of OxyHb

			and DeoxyHb.
Arterial blood gas	Provides reading of patient's oxygenation, ventilation, and metabolic condition. The specimen is obtained through an arterial puncture or catheter [1, 4].	• Samples can be arterial, venous, or a combination of both.	• Invasive and lacks ability to continuous monitoring [4].

References

[1] D. Castro, S. M. Patil, M. Zubair, and M. Keenaghan, "Arterial Blood Gas," in *StatPearls*. Treasure Island (FL), 2024.

[2] E. D. Chan, M. M. Chan, and M. M. Chan, "Pulse oximetry: understanding its basic principles facilitates appreciation of its limitations," (in eng), *Respir Med,* vol. 107, no. 6, pp. 789-99, Jun 2013, doi: 10.1016/j.rmed.2013.02.004.

[3] M. Nitzan, I. Nitzan, and Y. Arieli, "The Various Oximetric Techniques Used for the Evaluation of Blood Oxygenation," (in eng), *Sensors (Basel),* vol. 20, no. 17, Aug 27 2020, doi: 10.3390/s20174844.

[4] E. A. Abraham, G. Verma, Y. Arafat, S. Acharya, S. Kumar, and N. Pantbalekundri, "Comparative Analysis of Oxygen Saturation by Pulse Oximetry and Arterial Blood Gas in Hypoxemic Patients in a Tertiary Care Hospital," *Cureus,* vol. 15, no. 7, p. e42447, Jul 2023, doi: 10.7759/cureus.42447.

[5] P. Becker *et al.*, "Comparison of Hyperspectral Imaging and Microvascular Doppler for Perfusion Monitoring of Free Flaps in an In Vivo Rodent Model," *J Clin Med,* vol. 11, no. 14, Jul 16 2022, doi: 10.3390/jcm11144134.

[6] M. A. Calin, I. C. Boiangiu, S. V. Parasca, S. Miclos, D. Savastru, and D. Manea, "Blood oxygenation monitoring using hyperspectral imaging after flap surgery," (in English), *Spectroscopy letters,* vol. 50, no. 3, pp. 150-155, 2017, doi: 10.1080/00387010.2017.1297957.

[7] C. Sicher *et al.*, "Hyperspectral imaging as a possible tool for visualization of changes in hemoglobin oxygenation in patients with deficient hemodynamics – proof of concept," *Biomedical Engineering / Biomedizinische Technik,* vol. 63, no. 5, pp. 609-616, 2018, doi: doi:10.1515/bmt-2017-0084.

[8] M. Li, Y. Tang, and J. Yao, "Photoacoustic tomography of blood oxygenation: A mini review," (in eng), *Photoacoustics,* vol. 10, pp. 65-73, Jun 2018, doi: 10.1016/j.pacs.2018.05.001.
